# Supplementary material for: Association between Maternal Factors, Preterm Birth, and Low Birth Weight of Chilean Singletons
Source: Children (Basel). 2022 Jun 28;9(7):967. doi: 10.3390/children9070967 (PMC9319480; doi:10.3390/children9070967)
Supplement: Supplementary file 1 [file children-09-00967-s001.zip › children-1726412-supplementary.pdf]

Supplementary Materials:

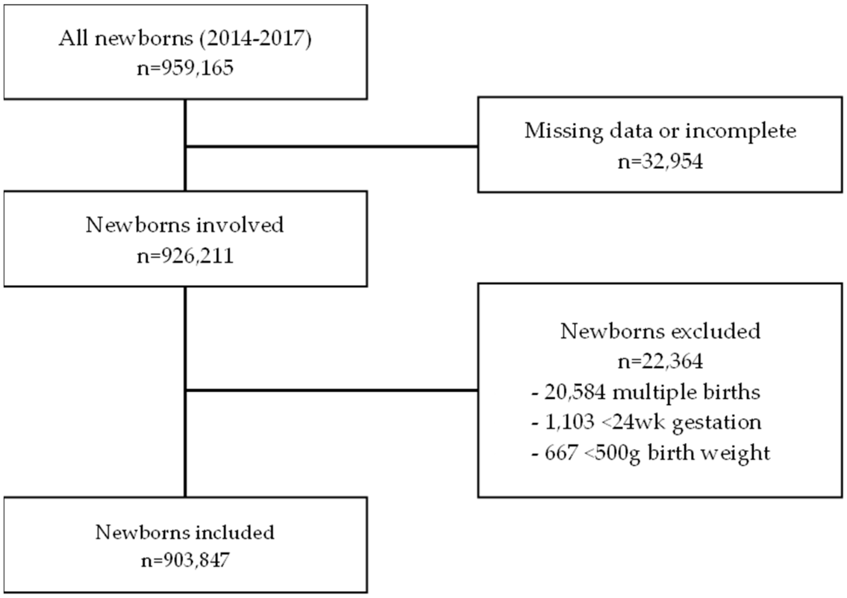

Figure S1. S. Flowchart of inclusion and exclusion.

Table S1. Equations for the PT and LWB model with maternal age > 40 years.

$logit\ (PT)$= -2.503 +0.456MA+0.176ME-0.029WM-0.081L-0.240MS-0.245S
$logit\ (LWB)$ =-2.951+0.462MA+0.206ME-0.046WM-0.094L-0.012MS-0.009S

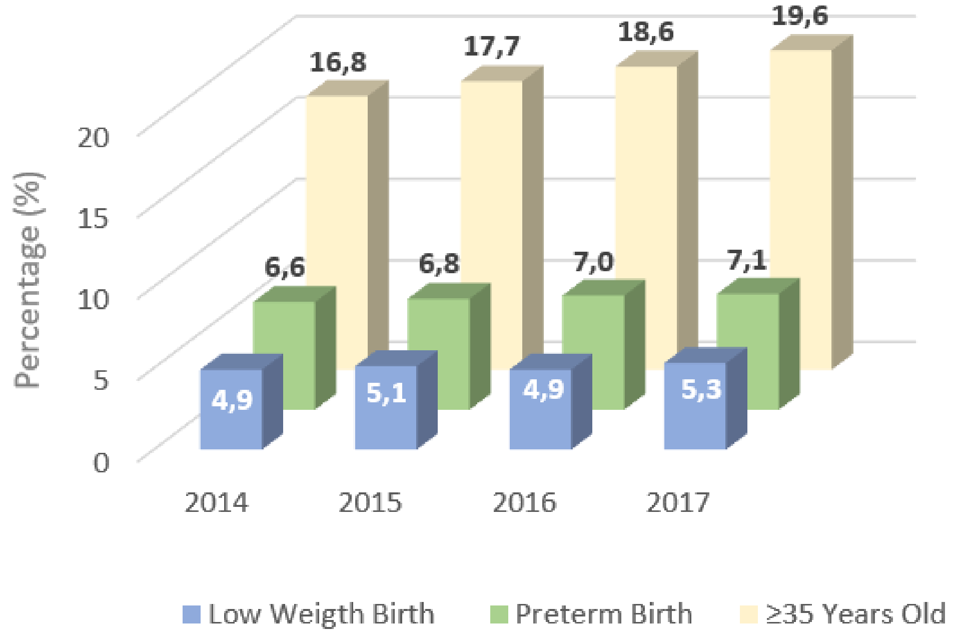

Figure S2. Annual increase in the prevalence of prematurity, low birth weight, and maternal age.

Table S2. Characteristics of newborns.

Variable	n=903,847	%
Prematurity		
Extremely preterm (<28wk)	2,558	0.3
Very preterm (≥28-<38wk)	5,918	0.6
Moderate o late preterm (≥32-<37wk)	53,388	5.9
Term (≥37-42wk)	841,983	93.2
Birth weight		
Extremely low birth weight (<1,000g)	2,728	0.3
Very low birth weight (≥1,000g-<1,500g)	4,554	0.5
Low birth weight (≥1,500g-<2,500g)	38,831	4.3
Normal (≥2,500g-<4,000g)	783,489	86.7
Macrosomia (≥4,000g)	74,245	8.2
Weight for gestational age		
Small for gestational age (SGA)	81,595	9.0
Appropriate for gestational age (AGA)	709,634	78.5
Large for gestational age (LGA)	112,618	12.5
Length for gestational age		
Small for gestational age (SGA)	128,974	14.3
Appropriate for gestational age (AGA)	714,014	79.0
Large for gestational age (LGA)	60,859	6.7

Table S3. Multiple logistic regression model for the association between preterm birth and low birth weight and maternal factors (maternal age > 40yo).

Variable	Preterm birth		Low birth weight	
	$OR_{crude\ (95\%\ CI)}$	$OR_{adjusted\ (95\%\ CI)}$	$OR_{crude\ (95\%\ CI)}$	$OR_{adjusted\ (95\%\ CI)}$
Maternal age (>40 years)	1.589 (1.535–1.645)	1.578 (1.524–1.634)	1.599 (1.537–1.664)	1.587 (1.535–1.651)
Educational attainment (<12 years)	1.214 (1.182–1.246)	1.192 (1.160–1.226)	1.259 (1.222–1.298)	1.229 (1.191–1.268)
Residence (urban)	0.946 (0.918–0.974)	0.922 (0.895–0.950)	0.941 (0.909–0.974)	0.911 (0.879–0.943)
Working mother	0.961 (0.946–0.977)	0.970 (0.954–0.9870)	0.942 (0.925–0.960)	0.955 (0.937–0.974)
Marital status (married or living with a partner)	0.977 (0.961–0.993)	0.976 (0.960–0.992)	0.989 (0.970–1.007)	0.988 (0.969–1.007)

Adjusted for sex.
